# Representation of Patients’ Hand Modulates Fear Reactions of Patients with Spider Phobia in Virtual Reality

**DOI:** 10.3389/fpsyg.2016.00268

**Published:** 2016-02-29

**Authors:** Henrik M. Peperkorn, Julia E. Diemer, Georg W. Alpers, Andreas Mühlberger

**Affiliations:** ^1^Department of Psychology I, University of WuerzburgWuerzburg, Germany; ^2^Clinical Psychology and Psychotherapy, Department of Psychology, University of RegensburgRegensburg, Germany; ^3^Department of Clinical and Biological Psychology and Psychotherapy, Mannheim School of Social Sciences, University of MannheimMannheim, Germany

**Keywords:** virtual reality, presence, immersion, perception, fear, specific phobia

## Abstract

Embodiment (i.e., the involvement of a bodily representation) is thought to be relevant in emotional experiences. Virtual reality (VR) is a capable means of activating phobic fear in patients. The representation of the patient’s body (e.g., the right hand) in VR enhances immersion and increases presence, but its effect on phobic fear is still unknown. We analyzed the influence of the presentation of the participant’s hand in VR on presence and fear responses in 32 women with spider phobia and 32 matched controls. Participants sat in front of a table with an acrylic glass container within reaching distance. During the experiment this setup was concealed by a head-mounted display (HMD). The VR scenario presented via HMD showed the same setup, i.e., a table with an acrylic glass container. Participants were randomly assigned to one of two experimental groups. In one group, fear responses were triggered by fear-relevant visual input in VR (virtual spider in the virtual acrylic glass container), while information about a real but unseen neutral control animal (living snake in the acrylic glass container) was given. The second group received fear-relevant information of the real but unseen situation (living spider in the acrylic glass container), but visual input was kept neutral VR (virtual snake in the virtual acrylic glass container). Participants were instructed to touch the acrylic glass container with their right hand in 20 consecutive trials. Visibility of the hand was varied randomly in a within-subjects design. We found for all participants that visibility of the participant’s hand increased presence independently of the fear trigger. However, in patients, the influence of the virtual hand on fear depended on the fear trigger. When fear was triggered perceptually, i.e., by a virtual spider, the virtual hand increased fear. When fear was triggered by information about a real spider, the virtual hand had no effect on fear. Our results shed light on the significance of different fear triggers (visual, conceptual) in interaction with body representations.

## Introduction

Phobic reactions can be triggered by specific perceptual (visual, tactile) cues and by fear-relevant conceptual information. In real life, both triggers coincide and activate an affective network (Lang, 1979; Bower, 1981; Foa and Kozak, 1986). Experimental studies on fear reactions show that perceptual, especially visual, cues are processed rapidly (Globisch et al., 1999) and with high specificity (Gerdes et al., 2009; Gerdes and Alpers, 2014), and result in typical activation patterns in the sympathetic nervous system. Besides, perceptual cues appear to be essential to trigger strong fear reactions in spider phobia (Peperkorn et al., 2014).

Studies on the power of verbal information to activate fear reactions mainly focus on the anticipation of threat (Melzig et al., 2007; Riemer et al., 2015). However, a few studies have investigated the effect of conceptual information in terms of propositional representations without presenting visual cues. Using the so-called instructed fear paradigm, Phelps et al. (2001) demonstrated that semantic information without the corresponding perceptual conditioned cues can induce stable fear reactions (Bublatzky et al., 2014). For VR, recent studies have demonstrated that both modalities (perception and conceptual information) constitute valid triggers of fear reactions (Bouchard et al., 2008; Gorini et al., 2011; Peperkorn et al., 2014), but so far research has mainly focused on visual perceptual cues.

In this context, virtual reality (VR) has proven useful as a means of investigating emotional processes, and as a new medium of exposure therapy for anxiety disorders (Mühlberger and Pauli, 2011; Diemer et al., 2015). The advantage of VR technology in the investigation of emotional reactions is that perceptual and conceptual information can be easily separated (Peperkorn et al., 2014; Shiban et al., 2016). Still, studies investigating the effects of tactile fear cues in VR are rare. Results from two case studies suggest that tactile cues intensify initial fear and the reduction of fear during exposure (Carlin et al., 1997; Hoffman et al., 2001). A controlled study on treatment efficacy of VR for spider phobia by Garcia-Palacios et al. (2002) confirms the impact of tactile cues. Usually, spider phobic patients report direct physical contact with a spider as extremely frightening.

Research on peripersonal space, i.e., the space around the body that is within the person’s reaching distance (Iachini et al., 2014), has shown that emotional processing is enhanced for stimuli in close proximity to the own body (Poliakoff et al., 2007; Åhs et al., 2015). These effects can be cross-modal, i.e., auditory (Taffou and Viaud-Delmon, 2014) and visual (Poliakoff et al., 2007) threat cues within peripersonal space have been found to enhance attention to tactile stimuli. Importantly, the effects of peripersonal space are also found in VR (Iachini et al., 2014; Åhs et al., 2015). However, the effects of combining perceptual (visual) vs. conceptual (verbal information) phobic stimuli with visual cues of physical proximity (a virtual presentation of one’s own hand) to these stimuli have not been investigated.

An important variable in VR is the sense of presence, i.e., the degree to which users feel involved in a VR world. Presence has been described as a necessary mediator that allows emotions to be elicited by an artificial, computer-generated scenario (Parsons and Rizzo, 2008; Price et al., 2011). Presence is commonly defined following Slater and Wilbur (1997, p. 605) as “the sense of being in the virtual environment” (Schubert et al., 2001). Findings of correlations between ratings of presence and fear confirm the importance of presence in VR research; however, the possible causal link between presence and emotions in VR has not yet been unraveled (Diemer et al., 2015; Peperkorn et al., 2015).

On the technological level, the more comprehensive the VR input to the sensory channels, i.e., the greater immersion, the more presence may be experienced (Slater and Wilbur, 1997; Slater, 1999). Consequently, the additional application of tactile cues intensifies and enriches presence (Hoffman et al., 1996). Further, self-focused attention can increase the salience of a situation and intensify emotional experience (Scheier and Carver, 1977). Earlier studies manipulated visual information of the own body with mirror images. Participants who saw their own body in a mirror reacted more intensely to emotionally relevant tasks than participants who did not see their own body (Phillips and Silvia, 2005).

In this study, we aimed to investigate the effects of the representation of the patients’ hand in VR as a way of including more reference to the own body in VR exposure. The paradigm is based on the phenomenon called the rubber hand illusion (RHI), which shows that – if the participant’s real hand is placed out of vision, and an artificial hand is shown instead – the participant can integrate the artificial hand as part of his/her own body (Botvinick and Cohen, 1998; Tsakiris and Haggard, 2005; Ehrsson et al., 2007; Longo et al., 2008; Riemer et al., 2015). The RHI has been successfully transferred into VR (Ijsselsteijn et al., 2006; Slater et al., 2009), and a similar procedure was chosen for the present study.

The goal of this study is to investigate if higher levels of self-representation intensify presence and fear in virtual phobia-relevant situations. In a previous study with patients with spider phobia, we found fear reactions to perceptual cues (seeing virtual spiders) to be more intense than fear in response to the information of the presence of a real spider (no spider visible in VR; Peperkorn et al., 2014). In the present study, we investigated the combined effects of perception of vs. information about the presence of a phobic stimulus and the presentation of a virtual hand (representing the patients’ real hand) on fear and presence. In accordance with research on the importance of peripersonal space and threat proximity (Poliakoff et al., 2007; Åhs et al., 2015), we expected fear reactions to increase when the virtual representation of the patients’ hand was included, especially in the perception condition (virtual spider visible). To test whether the virtual hand increased presence independently of fear, we included a control group without spider phobia.

## Materials and Methods

### Participants

Thirty-two women with spider phobia (age: 18–30 years; *M* = 22.75; *SD* = 2.72) and 32 healthy control participants, matched for age, completed this study. Participants were recruited with an online questionnaire which assessed inclusion criteria. These were for both groups: subjective fear rating of snakes below 15 (of 100), right-handedness, female gender, and age between 18 and 40 years. Additionally, the spider phobia group had to indicate a subjective fear rating of spiders above 75 (of 100) and fulfill diagnostic criteria of specific phobia according to DSM-IV. An additional inclusion criterion for control participants was subjective rating of fear of spiders below 15 (of 100). Exclusion criteria for both groups were a history of any psychiatric disorder (self-report; except for spider phobia in the phobia group). Patients and controls were randomly assigned to one of two experimental conditions (see below). The study was conducted in accordance with the Declaration of Helsinki and was approved by the local ethics committee (IRB of the medical school of the University of Würzburg). All participants gave written informed consent.

### Measures

#### Participant Characteristics

For baseline characteristics, a *demographic questionnaire* and the *State and Trait Anxiety Inventory, trait* form (STAI-t; Spielberger et al., 1970; German: Laux et al., 1981), were used. *Fear of snakes* was assessed with the Snake Anxiety Questionnaire (SNAQ; Klorman et al., 1974, German: Reinecke et al., 2009). Further, we applied the *Questionnaire on Disgust and Fear of Spiders* (German: Fragebogen zu Ekel und Angst vor Spinnen, FEAS; Schaller et al., 2006), which consists of the three subscales FEAS-fear, FEAS-disgust, and FEAS-somatic, to verify the expected differences between patients and controls regarding fear and disgust of spiders. For FEAS-fear and FEAS-disgust, participants are asked to rate the intensity of fear and disgust, respectively, they would experience in 14 situations concerning spiders (e.g., “You discover a spider on your leg”). Each item is scored on a 10-point Likert scale ranging from 0 (none at all) to 9 (very strong). For FEAS-somatic, participants rate the degree to which spiders cause each of 13 somatic symptoms (i.e., palpitations) on a 10-point Likert scale ranging from 0 (not at all applicable) to 9 (absolutely correct).

#### Process Measures

Verbal *self-reports of fear and presence* were rated on scales from 0 (not at all) to 100 (maximum). Fear was measured 10 and 40 s after the start of each exposure trial, presence after 20 and 50 s, respectively. *Skin conductance* was recorded throughout the experiment.

#### Outcome Measures

The following outcome measures were assessed before (pre) and after (post) the experimental session: *Fear of spiders* was assessed with the Fear of Spiders Questionnaire (FSQ; Szymanski and O’Donohue, 1995; German: Fragebogen zur Angst vor Spinnen; Rinck et al., 2002). In the FSQ participants rate how much 18 statements regarding spiders apply to them on a scale from 0 (not at all) to 6 (totally). For the measurement of *self-efficacy*, patients were asked to rate the likelihood that they would be able to rescue a trapped spider using a glass on a scale from 0 (not at all) to 100 (absolutely).

After exposure in VR, participants filled in the *Igroup Presence Questionnaire* (IPQ; Schubert et al., 2001). This instrument consists of 14 items and assesses presence on three scales, Spatial Presence (five items), Involvement (four items), and Realness (four items). An additional item measures General Presence. A 7-point Likert scale (0–6) was used for all items. For each scale, the mean score of the scale items is reported.

### Experimental Conditions

Participants were randomly assigned to one of two conditions, which differed in the type of fear trigger (perception vs. information).

#### Perception Condition

In VR, a laboratory with a spider in an acrylic glass container on a table (closely resembling the actual laboratory) was presented via a head-mounted display (HMD). Participants were informed that an acrylic glass container with a living *snake* in it (invisible to participants wearing the HMD) was placed on the real table in front of them, at the corresponding location where they saw the spider in VR.

#### Information Condition

Participants were informed that a living spider in an acrylic glass container was put in front of them. In VR, they saw a snake in a setting corresponding to the perception condition.

The living spider was a *Grammostola rosea* with a diameter of approximately 8 cm (including legs). The snake was a sub-adult *Pantherophis guttatus*, comparable in overall size to the spider. All animals were kept in a small acrylic glass container (23 cm × 15.3 cm × 16.5 cm) throughout the whole experiment. A snake was chosen as a control animal as it is comparable to spiders with regard to the concept of preparedness (Seligman, 1971; Öhman and Mineka, 2001), as both animals fall into the category of danger cues (Agras et al., 1969). For further information on the paradigm and the fear triggers (perceptual vs. information condition) see Peperkorn et al. (2014).

**FIGURE 1:**
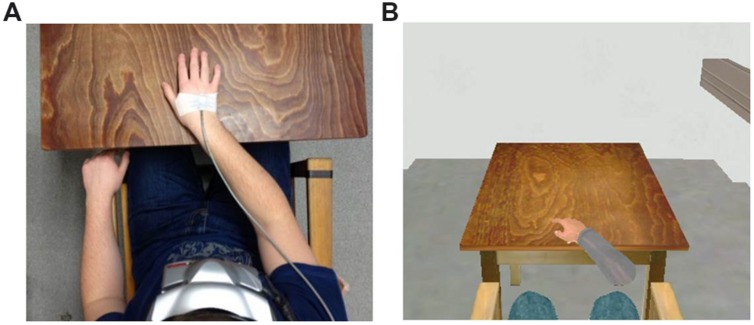
**Laboratory setup and corresponding VR scenario.** **(A)** Laboratory setup, including head-mounted display (HMD) and hand tracking. **(B)** VR scenario including VR hand. Phobic stimuli not shown.

#### Manipulation of Hand Visibility

Hand visibility in VR was realized via a virtual right hand and forearm. The position of the virtual hand was aligned to the participants’ right hand (see **Figure 1**). Movements (rotation and translation) of the virtual hand as a whole were tracked and presented in VR. Movements of individual fingers or the wrist were not rendered. The two levels of hand visibility (within subject manipulation: hand visible/hand not visible) were combined with both modalities of fear triggers (between subject manipulation: perception/information condition), resulting in four experimental conditions. All participants were instructed to touch the cover of the real acrylic glass container in front of them with their real hand in 2 × 10 consecutive exposure trials. In 50% of the trials the virtual hand was visible, while in the other 50% it was not. Presentation was in pseudo-randomized order, with no level of hand visibility displayed more than twice consecutively.

### Technical Equipment

An immersive 3-D VR environment was designed and programmed in vrml97 (Web3D Consortium). Additional 3-D elements were modeled and compiled with Autodesk Maya 2011 (Autodesk, Inc., San Rafael, CA, USA). The VR was rendered on a standard personal computer and displayed on a head mounted display (HMD, eMagin Z800 3DVisor, Bellevue, WA, USA). The experiment was controlled by the in-house built CyberSession Virtual Reality Interface (http://www.cybersession.info) running on a standard personal computer. A Polhemus 3space Fastrak position tracking system (6-DOF, Polhemus; Colchester, VT, USA) was used to measure head and hand positions. Acoustic instructions were supplied via headphones (HD 215, Sennheiser, Wedemark–Wennebostel).

For recordings of skin conductance, surface electrodes (Ag/AgCl; diameter: 13/6 mm) were attached to the second phalanx of the index finger and the middle finger of the left hand. Physiological signals were sampled and digitized with a Varioport-B system (Becker Meditec, Karlsruhe, Germany) and stored on a personal computer with a sampling frequency of 512 Hz.

### Procedure

Participants who met all criteria in the online questionnaire were invited to the study centre. At the screening session, all participants gave written informed consent, and a trained interviewer conducted the Structured Clinical Interview for DSM-IV (SCID; First et al., 1996; German: Wittchen et al., 1997) to rule out any psychiatric diagnoses in the control group and confirm the presence of spider phobia as the only diagnosis in the patient group. All participants were familiarized with the technical equipment and were immersed in the VR environment (without any phobic cues) for 5 min. They were trained in verbal self-reports (fear and presence). A second appointment was arranged and participants received written information as well as demographic and baseline questionnaires (STAI-t, SNAQ, FSQ, self-efficacy, FEAS) to be completed before the second appointment.

At the second appointment, the experimenter attached electrodes for skin conductance measurements and fitted the HMD and headphones. The experimental session in VR was delivered in two parts, each lasting approximately 21 min. The break between the two exposure blocks lasted at least 5, maximum 10 min, during which participants were asked to take off the HMD and headphones. At the beginning of the first exposure block, participants trained reaching for the cover of the acrylic glass container four times, without a time limit. The real container was empty and displayed congruently in VR. In two of the four training trials, the virtual hand was visible. A habituation phase (3 min) in the virtual laboratory followed. Then, participants completed a first block of ten consecutive exposure trials (each lasting 60 s). In each trial, a living spider or a snake in an acrylic glass container was placed on the table and displayed in VR according to the experimental condition. Participants were instructed to touch the cover of the acrylic glass container within the first 5 s after trial onset. Between each and after the last exposure trial, there was a pause of 30 s in which the real acrylic container was removed and not displayed in VR. The second part of the experiment in VR corresponded closely to the first part, with a habituation (3 min) followed by the second block of 10 consecutive exposure trials. The experiment concluded with a final recovery phase (3 min). The experimenter remained in the room during the entire experiment.

### Data Processing and Analysis

Physiological signals were filtered and segmented offline with the BrainVision Analyzer Software (Version 1.05, Brain Products Inc., Germany). Skin conductance reaction (SCR) was defined as a phasic response to the onset of an experimental trial in a response window of 1–10 s. Compared to conventional response windows of about 3–4 s (Dawson et al., 2007), this extended response window for SCR was chosen to account for the participants’ task at that time. The 3 s preceding each trial were used as baseline, and the maximum change in the response window relative to its corresponding baseline was computed for each trial. The mean SCR including zero responses was calculated to achieve an estimate of SCR magnitude (Dawson et al., 2007). Negative responses were set to zero. Mean SCR magnitude values were log-transformed [ln(SCR + 1)].

Baseline differences between groups were analyzed with two-way ANOVAs with the factors group (patients vs. controls) and fear trigger (perception vs. information). SCR, fear and presence ratings were analyzed with mixed ANOVAs with the between-subjects factors group (patients vs. controls) and fear trigger (perception vs. information), and the within-subject factors hand visibility (visible vs. invisible) and exposure block (first vs. second block). For measures assessed before and after exposure (FSQ, self-efficacy), we calculated difference scores, which were submitted to two-way ANOVAs with the between-subjects factors group (patients vs. controls) and fear trigger (perception vs. information).

All data analyses were performed with SPSS Statistics 21 (IBM Corp, USA). For all analyses, the confidence level was set to α = 0.05, and effect sizes for ANOVA reported as recommended by Tabachnick and Fidell (2007) as partial η^2^ ($\eta_{p}^{2}$) scores. For specific comparisons, Students *t*-tests were computed.

## Results

### Baseline Analyses

Baseline characteristics are shown in **Table 1.** As expected, fear of spiders (FEAS) was significantly stronger in patients than controls, as reflected in a significant main effect of group on the three subscales FEAS-fear (*F*_1,60_ = 268.93, *p* < 0.001, $\eta_{p}^{2}$ = 0.818), FEAS-disgust (*F*_1,60_ = 369.49, *p* < 0.001, $\eta_{p}^{2}$ = 0.860), and FEAS-somatic (*F*_1,60_ = 112.00, *p* < 0.001, $\eta_{p}^{2}$ = 0.651). Unexpectedly, there were significantly higher FEAS-disgust ratings in the perception condition (main effect fear trigger: *F*_1,60_ = 6.23, *p* = 0.015, $\eta_{p}^{2}$ = 0.094). Likewise, there was a trendwise interaction Group × Fear trigger for FEAS-somatic scores (*F*_1,60_ = 3.13, *p* = 0.082, $\eta_{p}^{2}$ = 0.050). *Post hoc F* tests revealed that this interaction was due to higher FEAS-somatic scores in the perception condition in controls only (*F*_1,30_ = 5.90, *p* = 0.021, $\eta_{p}^{2}$ = 0.164). There were no other significant effects on any of the FEAS scales. There were no significant differences between patients and controls, or between conditions regarding age, fear of snakes (SNAQ) or trait anxiety (STAI-t).

**Table 1:** Demographic and baseline measures of the study sample.

	Patients				Controls			
		
	Perception condition		Information condition		Perception condition		Information condition	
				
	*M*	*SD*	*M*	*SD*	*M*	*SD*	*M*	*SD*
Age	22.06	2.24	23.44	3.05	23.56	6.02	23.31	3.70
FEAS								
Fear	89.25	18.08	83.00	26.61	11.94	20.06	3.31	5.40
Disgust	106.81	11.18	97.50	16.95	28.00	21.38	16.56	15.35
Somatic reactions	35.13	13.60	43.31	22.21	5.69	5.30	2.06	2.74
SNAQ	4.44	2.06	5.00	3.86	4.50	2.80	4.13	2.42
STAI-t	32.19	6.75	36.75	8.50	37.88	7.54	35.44	12.47


*FEAS, Questionnaire on Disgust and Fear of Spiders; SNAQ, Snake Anxiety Questionnaire; STAI-t, State and Trait Anxiety Inventory, trait form.*

As for FSQ, ANOVA of baseline scored returned, as expected, a significant main effect of the factor group (*F*_1,60_ = 739.02, *p* < 0.001, $\eta_{p}^{2}$ = 0.925) due to higher scores in patients than in controls. ANOVA of self-efficacy ratings at baseline revealed, again as expected, a significant main effect of the factor group (*F*_1,60_ = 558.38, *p* < 0.001, $\eta_{p}^{2}$ = 0.903), with lower self-efficacy ratings in the patient group. There was also a significant main effect of the factor fear trigger (*F*_1,60_ = 6.84, *p* = 0.011, $\eta_{p}^{2}$ = 0.102), with higher self-efficacy ratings in the information condition (cf. **Table 2**).

**Table 2:** Presence and outcome questionnaire data of patients and non-anxious participants.

	Patients				Controls			
		
	Perception condition *n* = 16		Information condition *n* = 16		Perception condition *n* = 16		Information condition *n* = 16	
				
	*M*	*SD*	*M*	*SD*	*M*	*SD*	*M*	*SD*
IPQ								
General presence	4.13	1.31	4.25	1.57	2.69	1.30	3.00	1.46
Spatial presence	3.63	0.47	3.63	0.66	2.86	1.24	3.13	1.30
Involvement	2.88	0.90	2.22	0.65	1.91	1.16	1.95	0.90
Realism	3.56	0.77	2.94	0.70	2.23	1.08	2.11	0.93
FSQ								
Pre exposure	77.69	15.65	74.38	13.99	4.06	4.34	1.56	2.22
Post exposure	66.63	19.61	62.50	19.16	3.44	5.49	1.81	2.99
Self-efficacy								
Pre exposure	8.75	8.85	18.75	18.66	89.06	15.94	96.56	5.98
Post exposure	13.31	16.96	33.44	22.26	93.44	10.76	94.69	8.06


*IPQ, Igroup Presence Questionnaire; FSQ, Fear of Spiders Questionnaire.*

### Fear Ratings

**FIGURE 2:**
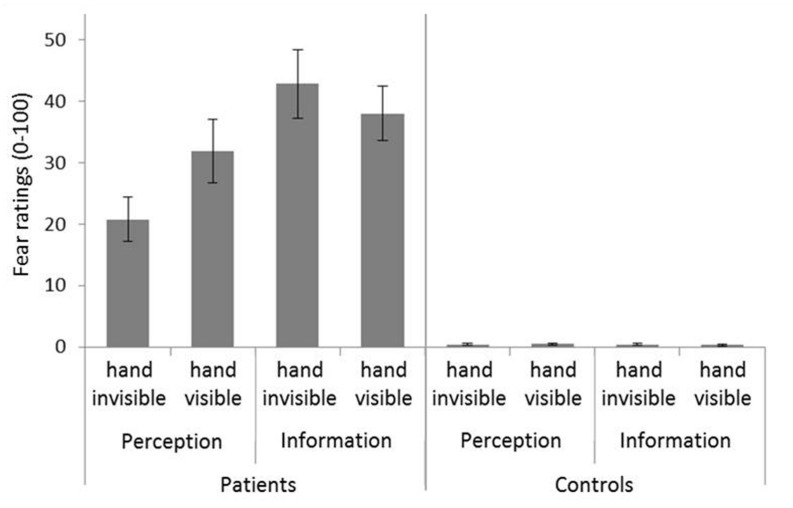
**Fear ratings.** Mean of all trials is shown. Error bars represent standard errors of the mean.

ANOVA of fear ratings over all participants confirmed significant main effects of exposure block (*F*_1,60_ = 92.13, *p* < 0.001, $\eta_{p}^{2}$ = 0.606), group (*F*_1,60_ = 118.43, *p* < 0.001, $\eta_{p}^{2}$ = 0.664), and fear trigger (*F*_1,60_ = 5.31, *p* = 0.025, $\eta_{p}^{2}$ = 0.081), as well as significant two-way interactions of Exposure block × Group (*F*_1,60_ = 85.93, *p* < 0.001, $\eta_{p}^{2}$ = 0.589) and Fear trigger × Group (*F*_1,60_ = 5.51, *p* = 0.022, $\eta_{p}^{2}$ = 0.084), and a significant three-way interaction of Hand visibility × Fear trigger × Group (*F*_1,60_ = 7.55, *p* = 0.008, $\eta_{p}^{2}$ = 0.112). This first analysis showed that as expected, controls reacted with much less fear than patients (see **Figure 2**). Consequently, to better understand the effects of the other factors in patients and controls, we conducted ANOVAs separately in each group.

In patients, there was a significant reduction of fear with time (main effect exposure block: *F*_1,30_ = 89.24; *p* < 0.001; $\eta_{p}^{2}$ = 0.748). Fear decreased from *M* = 41.78 (*SD* = 17.22) during the first exposure block to *M* = 25.00 (*SD* = 20.61) during the second exposure block. Further, there was a significant effect of fear trigger, with greater fear in the information condition (*F*_1,30_ = 5.42, *p* = 0.027, $\eta_{p}^{2}$ = 0.153). However, this latter effect was qualified by a significant interaction of Fear trigger × Hand visibility (*F*_1,30_ = 7.69; *p* = 0.009, $\eta_{p}^{2}$ = 0.204). There was no significant main effect of hand visibility. To follow up this interaction, we calculated separate ANOVAs with the factors hand visibility and exposure block for each fear trigger. Fear in the perception condition was significantly higher when the virtual hand was visible than when it was invisible (*F*_1,15_ = 5.26, *p* = 0.037, $\eta_{p}^{2}$ = 0.260). In the information condition, phobic patients’ fear did not differ significantly depending on hand visibility (see **Figure 2**).

In controls, ANOVA returned a significant main effect of exposure block (*F*_1,30_ = 10.47, *p* = 0.003, $\eta_{p}^{2}$ = 0.259), indicating a decrease of generally very low fear ratings over time. Fear decreased from *M* = 2.70 (overall mean; *SD* = 0.53) during the first exposure block to *M* = 1.85 (*SD* = 0.23) during the second exposure block. Neither the effects of hand visibility, nor of fear trigger reached significance.

To check whether these results were influenced by the (unexpected) baseline difference on the FEAS-disgust scale between fear trigger conditions (see above), we repeated the ANOVA with the FEAS disgust baseline score as a covariate. The inclusion of this covariate did not change the pattern of results.

### Presence Ratings

**FIGURE 3:**
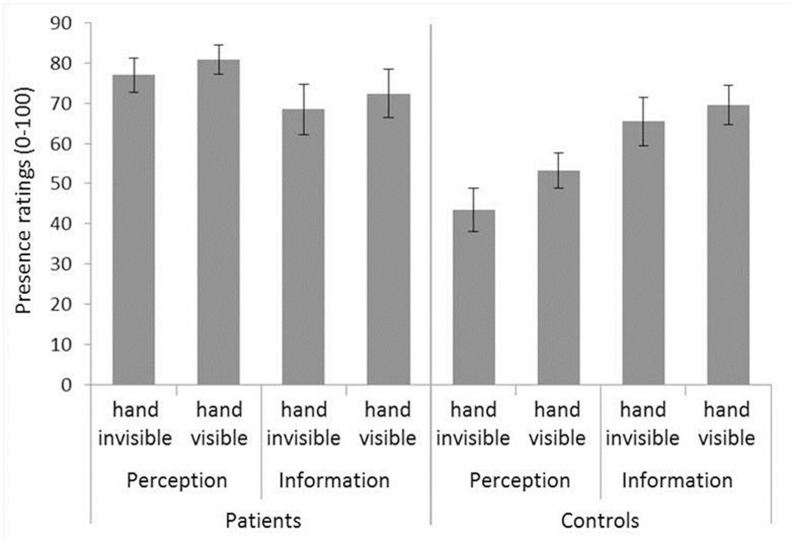
**Presence ratings.** Mean of all trials is shown. Error bars represent standard errors of the mean.

ANOVA of presence ratings revealed significant main effects of the factors hand visibility (*F*_1,60_ = 36.38, *p* < 0.001, $\eta_{p}^{2}$ = 0.377), indicating a more intensive experience of presence when participants saw a digital representation of their hand in VR, and exposure block (*F*_1,60_ = 7.21, *p* = 0.009, $\eta_{p}^{2}$ = 0.107), due to a linear decrease of presence throughout exposure trials, and group (*F*_1,60_ = 10.71, *p* = 0.002, $\eta_{p}^{2}$ = 0.151), with patients experiencing significantly more presence than controls (see **Figure 3**). There was a trendwise interaction of Exposure block × Group (*F*_1,60_ = 3.44, *p* = 0.069, $\eta_{p}^{2}$ = 0.054): Patients reported a mean presence of *M* = 76.90 (*SD* = 19.78) during the first exposure block, and *M* = 72.40 (*SD* = 22.41) during the second exposure block. The presence values for controls were *M* = 58.35 (*SD* = 21.33), and *M* = 57.53 (*SD* = 23.57) for the first and second exposure blocks, respectively. There was no significant main effect of the factor fear trigger, however, the Group × Fear trigger interaction was significant (*F*_1,60_ = 7.33, *p* = 0.009, $\eta_{p}^{2}$ = 0.109). To follow up the significant Group × Fear trigger interaction, we conducted ANOVAs separately in each group.

In patients, there was no significant difference between fear triggers. Controls, however, experienced significantly more presence in the information than the perception condition (*F*_1,30_ = 7.04; *p* = 0.013; $\eta_{p}^{2}$ = 0.190).

### Skin Conductance Reaction

**FIGURE 4:**
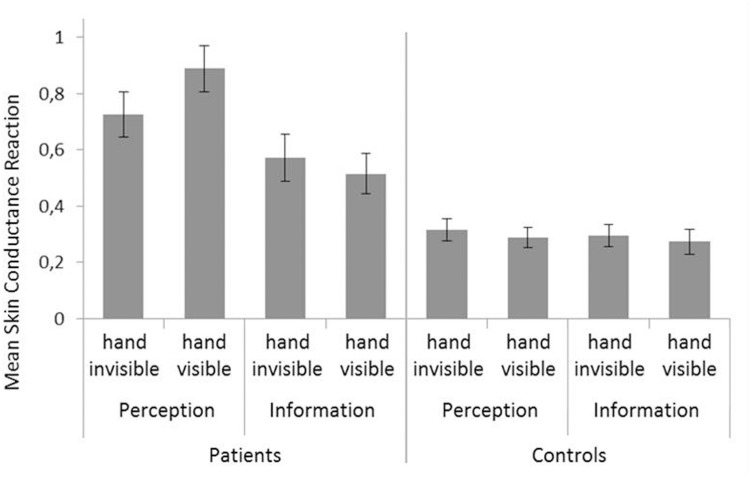
**Skin Conductance Reactions (SCR).** Mean of all trials is shown (log-transformed data; SCR magnitude). Error bars represent standard errors of the mean.

ANOVA of SCR showed significant main effects of exposure block (*F*_1,60_ = 17.36, *p* < 0.001, $\eta_{p}^{2}$ = 0.224) indicating a decrease with time, group (*F*_1,60_ = 41.88, *p* < 0.001, $\eta_{p}^{2}$ = 0.411), due to higher SCR in patients, and fear trigger (*F*_1,60_ = 5.75; *p* = 0.020, $\eta_{p}^{2}$ = 0.087), indicating higher SCR in the perception than the information condition (see **Figure 4**). However, these effects were qualified by significant two-way interactions of Hand visibility × Fear trigger (*F*_1,60_ = 6.16, *p* = 0.016, $\eta_{p}^{2}$ = 0.093), and Fear trigger × Group (*F*_1,60_ = 4.33, *p* = 0.042, $\eta_{p}^{2}$ = 0.067), and a significant three-way interaction of Hand visibility × Fear trigger × Group (*F*_1,60_ = 6.84, *p* = 0.011, $\eta_{p}^{2}$ = 0.102). To clarify the sources of these interaction effects, separate ANOVAs were calculated for patients and controls.

In patients, there were significant main effects of the factor exposure block (*F*_1,30_ = 7.84, *p* = 0.009, $\eta_{p}^{2}$ = 0.207), due to a decrease with time: SCR decreased from *M* = 0.77 (overall mean; *SD* = 0.36) during the first exposure block to *M* = 0.59 (*SD* = 0.38) during the second exposure block. There was also a significant effect of the factor fear trigger (*F*_1,30_ = 6.28, *p* = 0.018, $\eta_{p}^{2}$ = 0.173), indicating higher SCR in the perception condition. However, this latter effect was qualified by a significant interaction of Hand visibility × Fear trigger (*F*_1,30_ = 7.95, *p* = 0.008, $\eta_{p}^{2}$ = 0.209; see **Figure 4**). *Post hoc* pair-wise comparisons showed that there was a significant effect of hand visibility only in the perception condition (*F*_1,30_ = 8.661, *p* = 0.006, $\eta_{p}^{2}$ = 0.224), where SCR was higher when the patient’s hand was visible.

In controls, there was only a significant main effect of the factor exposure block (*F*_1,30_ = 20.60, *p* < 0.001, $\eta_{p}^{2}$ = 0.407, due to a decrease of SCR with time from *M* = 0.35 (*SD* = 0.16) during the first to *M* = 0.24 (*SD* = 0.16) in the second exposure block.

### Questionnaire Data

#### Igroup Presence Questionnaire (IPQ)

Univariate analyses of variance with the factors group and fear trigger were calculated for general presence and the three subscales of the IPQ. Results revealed significantly higher scores for patients in general presence (*F*_1,60_ = 14.42, *p* < 0.001, $\eta_{p}^{2}$ = 0.194), and for all three IPQ subscales: spatial presence (*F*_1,60_ = 6.57, *p* = 0.013, $\eta_{p}^{2}$ = 0.099), involvement (*F*_1,60_ = 7.19, *p* = 0.009, $\eta_{p}^{2}$ = 0.107), and realism (*F*_1,60_ = 23.91, *p* < 0.001, $\eta_{p}^{2}$ = 0.285). No significant effect of the factor fear trigger was detected.

#### Fear of Spiders Questionnaire (FSQ)

ANOVA of FSQ difference scores returned only a main effect of the factor group (*F*_1,60_ = 20.37, *p* < 0.001, $\eta_{p}^{2}$ = 0.253) due to a greater reduction of FSQ scores in patients compared to controls (cf. **Table 2**).

#### Self-efficacy

ANOVA of self-efficacy difference scores revealed a significant main effect of the factor group (*F*_1,60_ = 5.56. *p* = 0.022, $\eta_{p}^{2}$ = 0.085), due to a greater change in self-efficacy in patients vs. controls. However, this effect was qualified by a significant interaction Group × Fear trigger (*F*_1,60_ = 5.31, *p* = 0.025, $\eta_{p}^{2}$ = 0.081). *Post hoc F* tests showed that only in patients, there was a significantly greater increase in self-efficacy in the information condition (*F*_1,60_ = 4.06, *p* = 0.048, $\eta_{p}^{2}$ = 0.063). In controls, the increase of self-efficacy did not differ significantly between conditions (cf. **Table 2**).

## Discussion

We exposed patients with spider phobia and matched healthy controls repeatedly to a VR scenario where they touched a transparent container. We varied (a) whether a spider was present only in VR (perception condition) or only in the real lab (unseen by participants; information condition), and (b) whether participants saw a representation in VR of their own hand, or not. As expected, the representation of the participants’ hand within VR increased presence in patients with spider phobia and in healthy controls. Importantly, in patients, the influence of this visibility on fear depended on the fear trigger. When fear was triggered perceptually (by a virtual spider), it was higher if a representation of the hand was also visible. When fear was triggered by information about a real spider, the virtual representation of the hand did not influence fear reactions. To our knowledge, this is the first study to demonstrate the interaction between representation of the participants’ body (here: hand), and different fear triggers (perception vs. information).

We found that presence increased in both groups and with both fear triggers when the hand was visible in VR. This result is in line with research on immersion and presence, which generally finds that greater immersion is related to enhanced presence (Diemer et al., 2015). For example, more sophisticated presentation, like HMD vs. computer monitor (Gorini et al., 2011), or stereoscopy vs. monoscopy (Ijsselsteijn et al., 2001; Ling et al., 2012; Peperkorn et al., 2015) lead to increased presence. Augmenting VR by haptic stimulation (e.g., touching a toy spider) has also been shown to increase presence (Hoffman et al., 2003; Peperkorn and Mühlberger, 2013). Overall, we found rather high presence ratings in both phobic patients and control participants, but presence was still significantly higher in patients, which experienced fear, while controls reported almost no fear. Higher presence in patients was evident consistently both in the verbal ratings during exposure, as well as the post-exposure presence questionnaire (IPQ). This pattern corresponds well with the interoceptive attribution model of presence proposed by Diemer et al. (2015): While immersion leads to a basic level of presence, highest levels of presence are reached only when emotions are engaged, as is the case for the patients. This leads to the assumption that basic presence, which is triggered independent of emotional relevance by the immersive nature of a VR setup, might be a prerequisite for emotional engagement, which in turn intensifies presence.

The finding that, in patients, fear was enhanced by the VR hand in the perception condition only (where the spider was visible in VR) is in line with findings on the significance of peripersonal space for fear. It is known that fearful stimuli within peripersonal space increase the allocation of attention to this area (Poliakoff et al., 2007), and that emotional processing is enhanced for stimuli within peripersonal space, as indexed by greater startle response and more stable fear conditioning (Åhs et al., 2015). Seeing one’s hand in close proximity to the virtual spider while touching an (external) enclosure in the same position firmly establishes the fearful stimulus as within the virtual peripersonal space. Not seeing the hand while touching that enclosure renders the situation rather ambiguous with regard to peripersonal space, as real and virtual peripersonal space are dissociated. Following this, a VR representation of the hand might be used to manipulate peripersonal space, as the virtual hand is assimilated as belonging to the self. To what extent this effect depends on the realism of the virtual hand is open to investigation. A recent review on the “uncanny valley effect” of virtual stimuli suggests that for moving stimuli (like our VR hand), naturalistic movement is more important than realistic visuals (de Borst and de Gelder, 2015), but clearly more research is needed.

In the unambiguous condition (both the spider and the hand are visible in VR), fear in patients was higher than in the ambiguous situation (the VR spider, but not the hand are visible). This effect may also be explained by emotional context. Studies on emotional perception for mixed scenes (foreground and background of different or matching emotional valence; Kret and de Gelder, 2010; Van den Stock et al., 2014a,b) or multimodal emotional cues (i.e., sounds and pictures; Gerdes et al., 2014) have found that emotion recognition is influenced by the interaction of multimodal emotional content. For example, participants are slower to recognize a neutral vs. fearful body expression when the background is threatening (Van den Stock et al., 2014a), and are faster at recognizing bodily expressions of emotion if these are shown against a matching emotional (social) scene (Kret and de Gelder, 2010). In our study, the combination of the visible, “threatened” hand (rather than an invisible, “safe” hand) and the visible spider is emotionally unambiguous and goes along with a stronger emotional reaction. However, it remains to be tested whether the facilitating effect of emotional congruence observed in emotion recognition studies (Kret and de Gelder, 2010; Van den Stock et al., 2014a,b) translates to emotional experience, as measured in our study.

Unexpectedly, patients experienced more fear in the information than the perceptual condition, i.e., when they knew that they were touching – outside VR – a container with a real life spider in it. In previous studies, we found the opposite pattern, with significantly higher subjective fear in the conditions including perceptual fear triggers in spider phobia (Peperkorn et al., 2014). However, in contrast to the study by Peperkorn et al. (2014), in the present study patients in the information condition touched the container with a life spider in it. The distance between spider and participants was much smaller and participants had to actively approach the fear trigger in this study. Touching the container provided haptic stimulation that indicated the physical presence of the feared object. This aspect renders the information condition of this study similar to the haptic stimulation condition applied by Peperkorn and Mühlberger (2013), where participants saw a VR spider on their hand, or felt a spider dummy in the same place, or experienced a combination of both. Peperkorn and Mühlberger (2013) found higher fear levels in the conditions including haptic stimulation vs. only visual exposure. Haptic information may lead to a shift in attentional focus, only when visual material is focused on, it will fully activate the fear network (Alpers et al., 2009).

The results of the present study reproduce the common fear hierarchy of patients with spider phobia, where touching a spider is most aversive, and seeing one is usually more fear-inducing than knowing that a hidden spider is present. The results of the present study may also be explained in the light of the importance of peripersonal space for fear processing, as haptic stimulation is the most direct indicator that the feared object is within peripersonal space. Our results suggest that in direct comparison, a real feared object within real peripersonal space is more fear-inducing than a VR object within virtual peripersonal space.

In some respect, SCRs parallel findings of fear ratings. This especially holds true for differences in experienced fear in the perception condition. Seeing one’s own hand within the VR next to a virtual spider leads to higher SCR than when the hand is not displayed. In the information condition, by contrast, there was no effect of hand visibility. Interestingly, SCRs were lower overall in the information condition than in the perception condition. Subjective and physiological reactions dissociate in this respect. However, differences between subjective and physiological reactions toward fear cues are not uncommon (Hermans et al., 2005). Our findings suggest that information may trigger fear reactions through cognitive processing of this information. In contrast, visual cues trigger automatic reactions toward this cue. This may reflect the two routes of fear described by LeDoux (1996), i.e., fear as the expression of a basic, unconscious “survival function[s]” (LeDoux, 2012, p. 654) vs. conscious responses (LeDoux, 2014).

Although total exposure time to spider stimuli was just 20 min, we found a significant increase of self-efficacy, and a corresponding reduction in fear of spiders (FSQ score) in patients. These results correspond to a significant reduction already during the exposure session of the online measures of fear and arousal (subjective fear ratings and SCR). In view of the VR literature, decreases in subjective fear and fear questionnaires have been reported for single sessions in specific phobia (Mühlberger et al., 2006; Shiban et al., 2013, 2015) as well as a short course of three short sessions for fear of speaking (Harris et al., 2002). Likewise, the physiological reactivity to fear situations has been reported to drop over similar time frames (Harris et al., 2002; Mühlberger et al., 2007; Shiban et al., 2013). Most studies on therapy effects of VR exposure have, however, applied longer treatment protocols, so more research is needed to understand the subjective and physiological changes that occur in one session exposure treatments and during the initial states of multi-session VR exposure therapy.

Some limitations of our study should be taken in to account. First, our study sample was composed of women only, so generalization of our results to men, or indeed to other anxiety disorders, is impossible. Despite randomization, there were small, unexpected differences between fear trigger conditions on the FEAS disgust subscale (patients and controls) and the FEAS somatic symptoms subscale (controls only). However, these differences seem not to have influenced results. Further, our study design included a series of twenty brief exposure trials (60 s each), so results are not necessarily comparable to outcomes from studies that applied longer exposure durations. While we found a decrease in subjective fear ratings, fear of spiders, and SCR with time, as well as an increase in self-efficacy after exposure in patients, possibly indicating the beginning of therapeutic exposure effects, our design does not allow conclusions about therapy processes. Neither did our participants receive any treatment rationale for exposure or any form of cognitive preparation, which is usually part of exposure therapy.

Our results shed light on the impact of different fear triggers (visual, conceptual) in interaction with body representations on subjective fear, presence, and physiological arousal. Our results further show that proper planning of a virtual scenario is essential. By activating the fear network via combined visual and tactile triggers, we were able to induce high fear intensities. Further research is needed to determine the function of different fear triggers, their different impact on fear reactions dependent on factors like distance or active behavior, and the relation of fear triggers to different routes of fear processing (LeDoux, 2012, 2014).

## Authors Contributions

HP, GA, and AM designed the study. HP recruited the participants and acquired the data. HP, JD, and AM analyzed and interpreted the data. HP and JD drafted the manuscript. All authors critically revised the manuscript, approve of the final version, and agree to be accountable for its content.

## Conflict of Interest Statement

The authors declare that the research was conducted in the absence of any commercial or financial relationships that could be construed as a potential conflict of interest.

AM is stakeholder of a commercial company that develops virtual environment research systems.
